# Supplementary material for: Glycation Interferes with the Expression of Sialyltransferases and Leads to Increased Polysialylation in Glioblastoma Cells
Source: Cells. 2023 Dec 2;12(23):2758. doi: 10.3390/cells12232758 (PMC10706364; doi:10.3390/cells12232758)
Supplement: Supplementary file 1 [file cells-12-02758-s001.zip › 2023_11_08 Supplementary Material.pdf]

Supplementary Material

Table S1: Genetic background of glioma cell lines.

Cell line	U251	LN229	U343 (Grad III / Grad IV)
**Gender, Age**	Male, 75 years	Female, 60 years	Male, 54 years
**Known driver mutations** [1],[2]	*CDKN2a* del<br>*PTEN* mut<br>*p53* mut<br>*IDH1/2* wt<br>*NF1* mut	*CDKN2a* del<br>*PTEN* wt<br>*p53* mut<br>*IDH1/2* wt	*CDKN2a* del<br>*PTEN* mut<br>*p53* wt<br>*NF1* mut<br>*IDH1/2* wt
**Subtype**	mesenchymal	proneural	n.a.

Abbreviations: mut: mutation; wt: wild-type; del: deletion; *CDKN2a: cyclin-dependent kinase inhibitor 2A; PTEN: Phosphatase and Tensin Homolog deleted on Chromosome 10; p53: protein 53; IDH1/2: Isocitrate dehydrogenase 1/2; NF1: Neurofibromatosis 1*

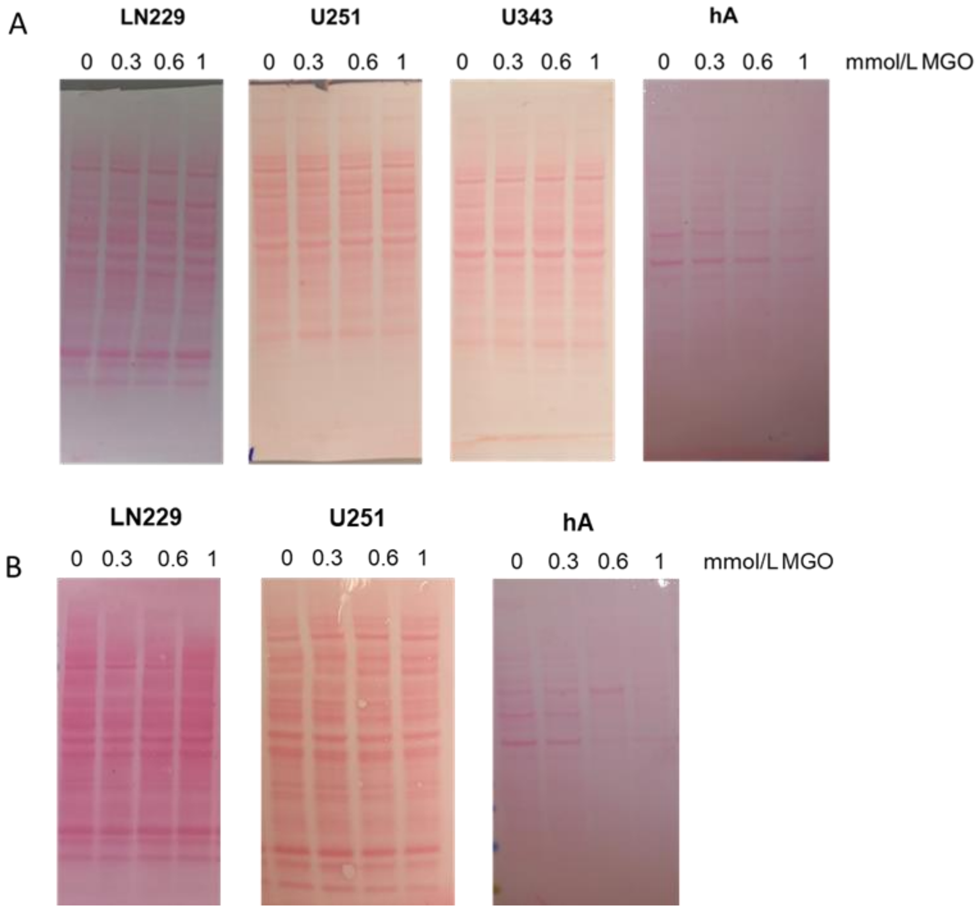

Figure S1: Ponceau S staining. Ponceau S staining was performed on protein bands of LN229, U251, U343 and hA treated with various concentrations of MGO (0, 0.3, 0.6, and 1 mmol/L). The Ponceau S staining (A) corresponds to the Western blots illustrated in Figure 5 and the Ponceau S staining (B) corresponds to the Western blots illustrated in Figure 10.

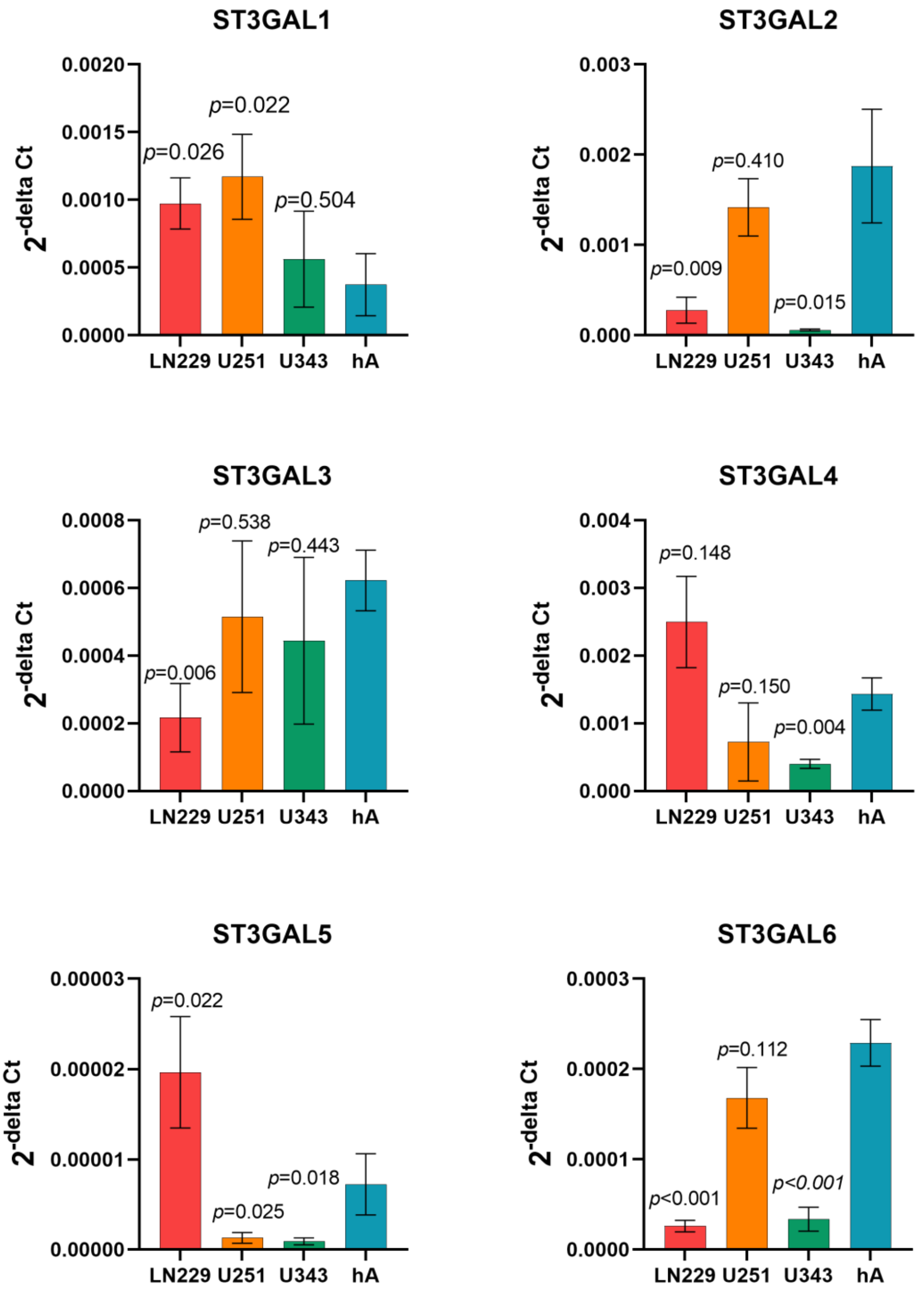

Figure S2: mRNA levels of ST3GAL family in glioma cell lines compared to hA. Graphs show mRNA level of ST3GAL1-6 in LN229 cells, U251 cells, U343 cells and hA, measured with qPCR. Student's t-test was performed for statistical analysis. *P*-values represent the comparison of glioma cell lines (LN229, U251, U343) mRNA level with hA. Presented are means and SD of four independent biological replicates.

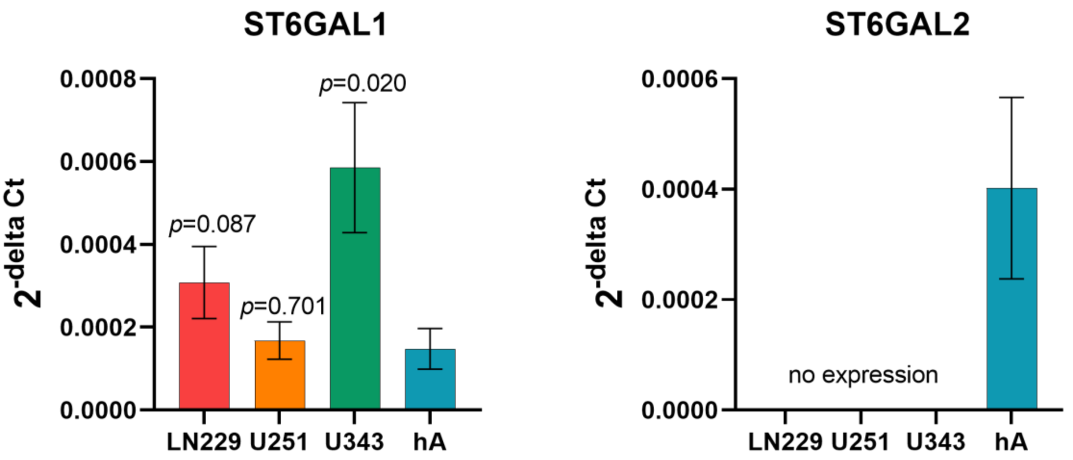

Figure S3: mRNA levels of ST6GAL family in glioma cell lines compared to hA. Graphs show mRNA level of ST6GAL1 and 2 in LN229 cells, U251 cells, U343 cells and hA, measured with qPCR. Student's t-test was performed for statistical analysis. *P*-values represent the comparison of glioma cell lines (LN229, U251, U343) mRNA level with hA. Presented are means and SD of four independent biological replicates.

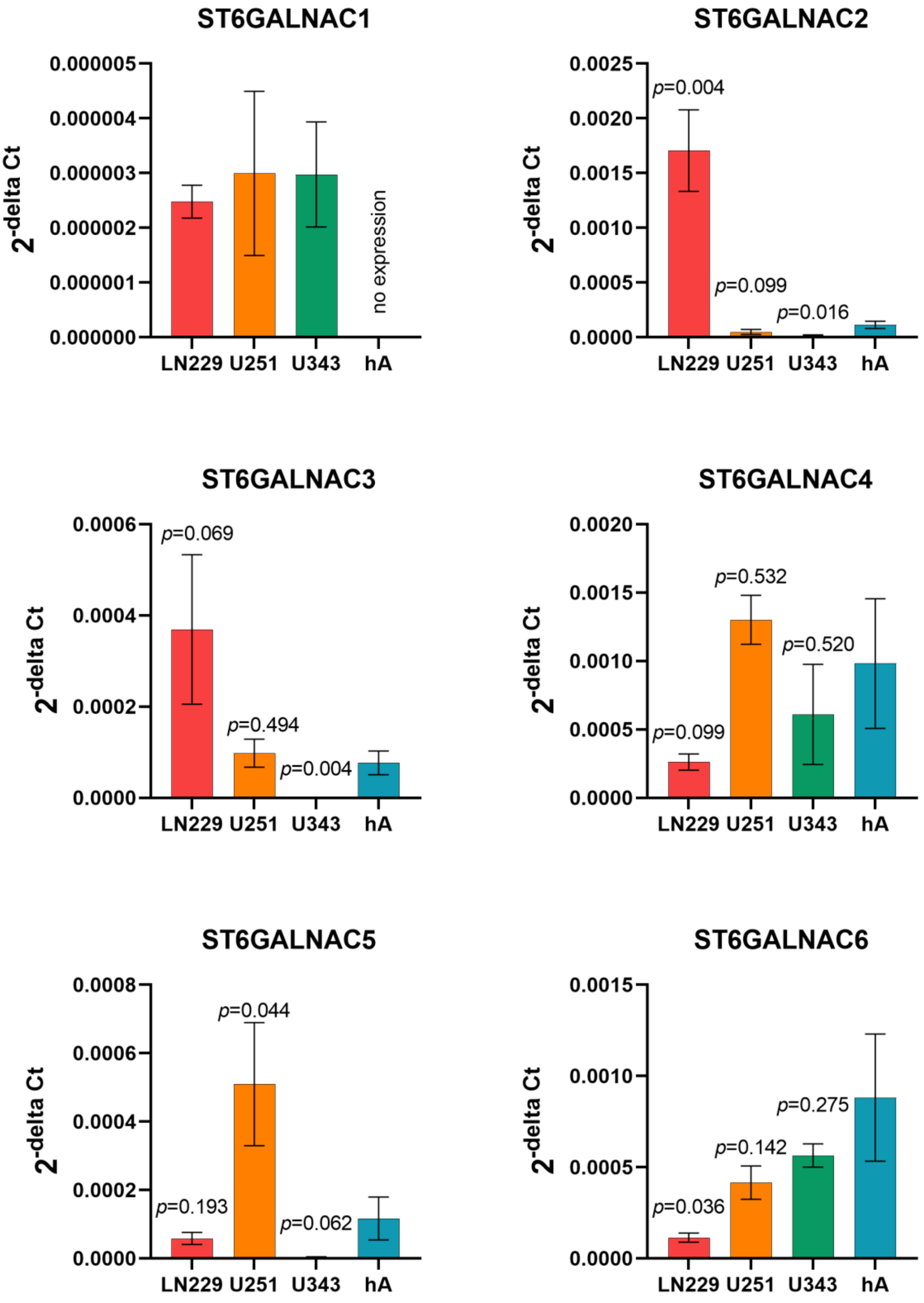

Figure S4: mRNA levels of ST6GALNAC family in glioma cell lines compared to hA. Graphs show mRNA level of ST6GALNAC1-6 in LN229 cells, U251 cells, U343 cells and hA, measured with qPCR. Student's t-test was performed for statistical analysis. *P*-values represent the comparison of glioma cell lines (LN229, U251, U343) mRNA level with hA. Presented are means and SD of four independent biological replicates.

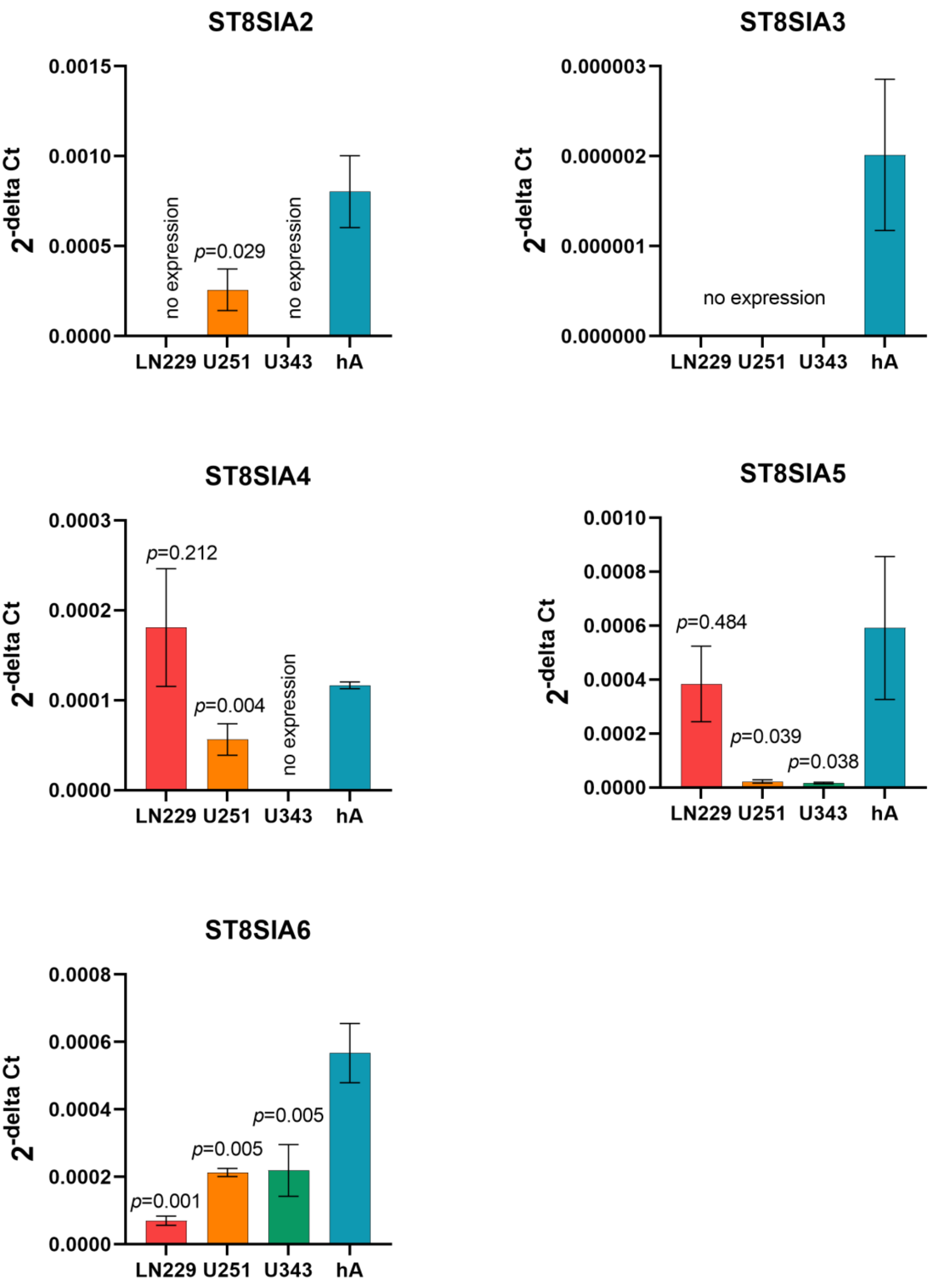

Figure S5: mRNA levels of ST8SIA family in glioma cell lines compared to hA. Graphs show mRNA level of ST8SIA2-6 in LN229 cells, U251 cells, U343 cells and hA, measured with qPCR. Student's t-test was performed for statistical analysis. *P*-values represent the comparison of glioma cell lines (LN229, U251, U343) mRNA level with hA. Presented are means and SD of four independent biological replicates.

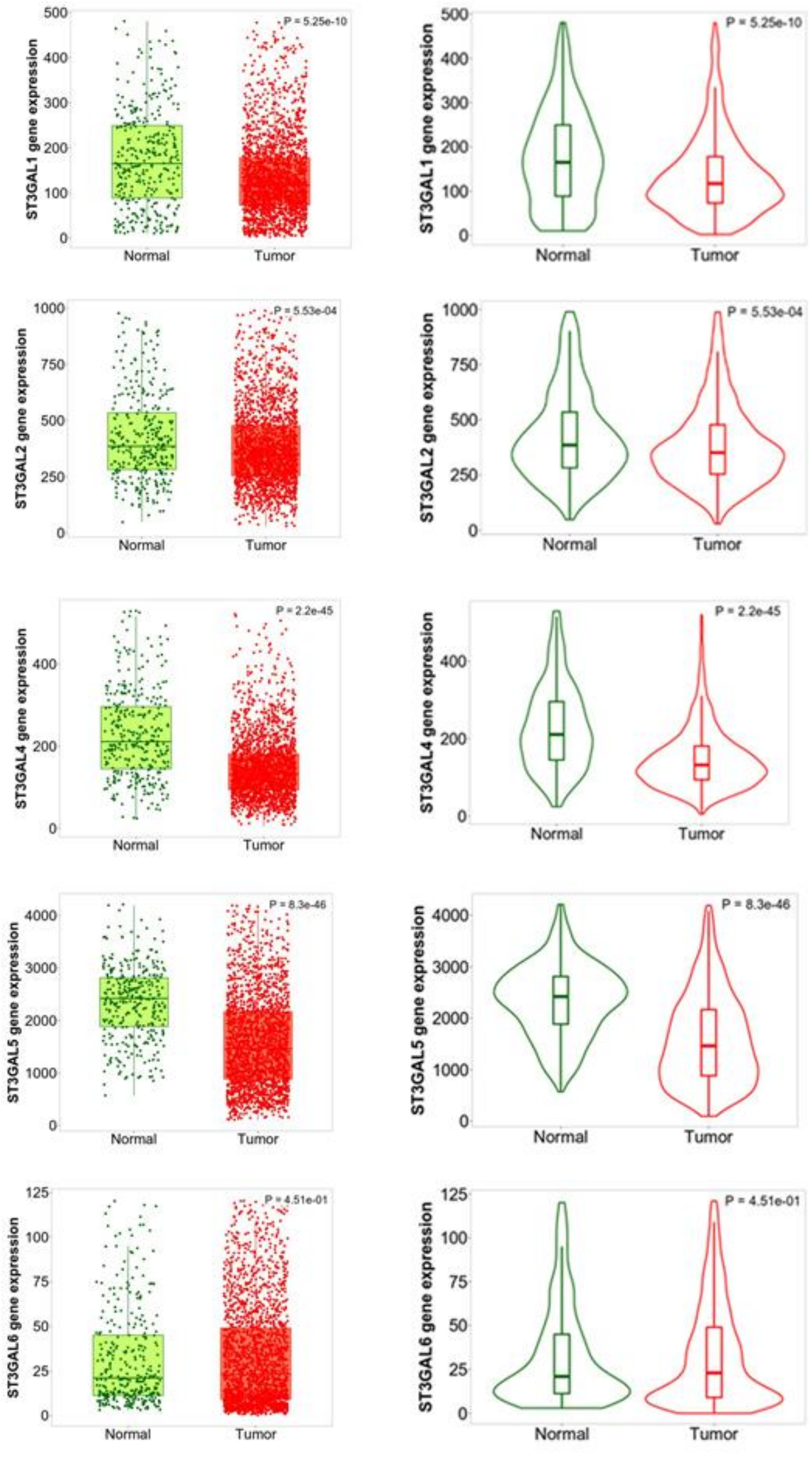

Figure S6: ST3GAL family gene expression in CNS tumors and normal tissue. Boxplots (left) and violin plots (right) of ST3GAL family gene expression in CNS tumors when comparing paired normal (n=339) and tumor (n=2159) microarray data. Comparisons were performed using the Mann-Whitney U test with a statistical significance threshold set at p value <0.01. Data was obtained from TNMplot (TNMplot.com last access date 03/19/2023).

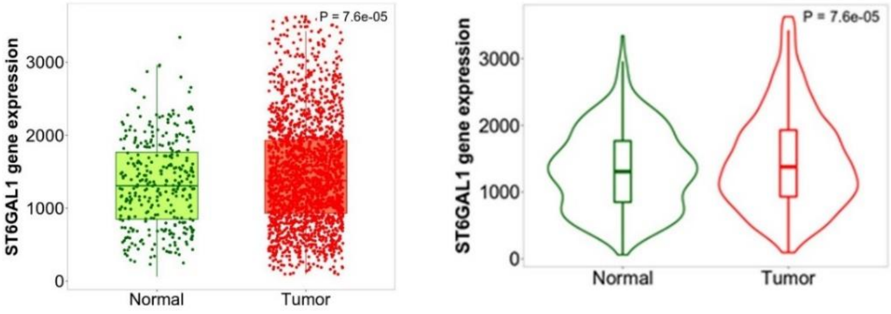

Figure S7: ST6GAL family gene expression in CNS tumors and normal tissue. Boxplots (left) and violin plots (right) of ST6GAL family gene expression in CNS tumors when comparing paired normal and tumor microarray data. Comparisons were performed using the Mann-Whitney U test with a statistical significance threshold set at p value <0.01. Data was obtained from TNMplot (TNMplot.com last access date 03/19/2023).

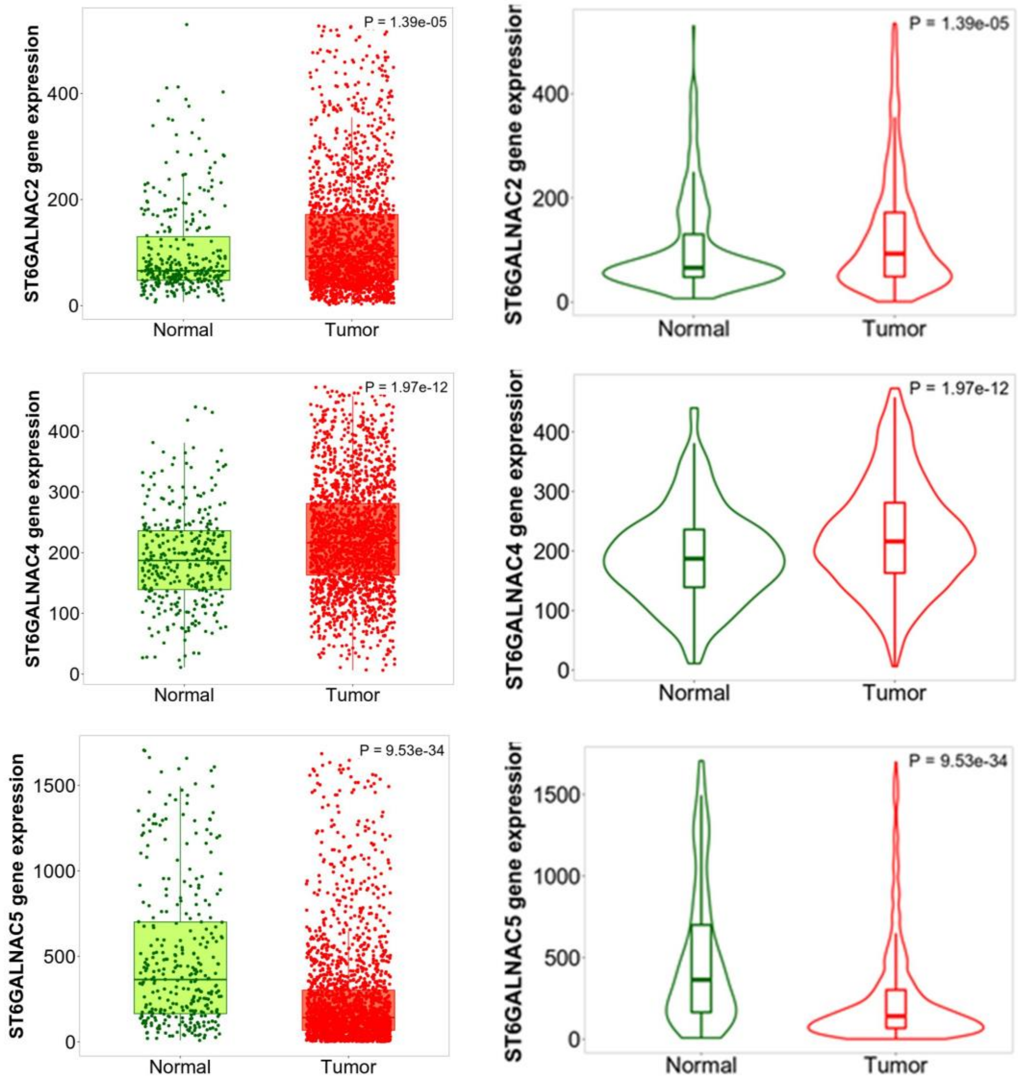

Figure S8: ST6GALNAC family gene expression in CNS tumors and normal tissue. Boxplots (left) and violin plots (right) of ST6GALNAC family gene expression in CNS tumors when comparing paired normal and tumor microarray data. Comparisons were performed using the Mann-Whitney U test with a statistical significance threshold set at p value <0.01. Data was obtained from TNMplot (TNMplot.com last access date 03/19/2023).

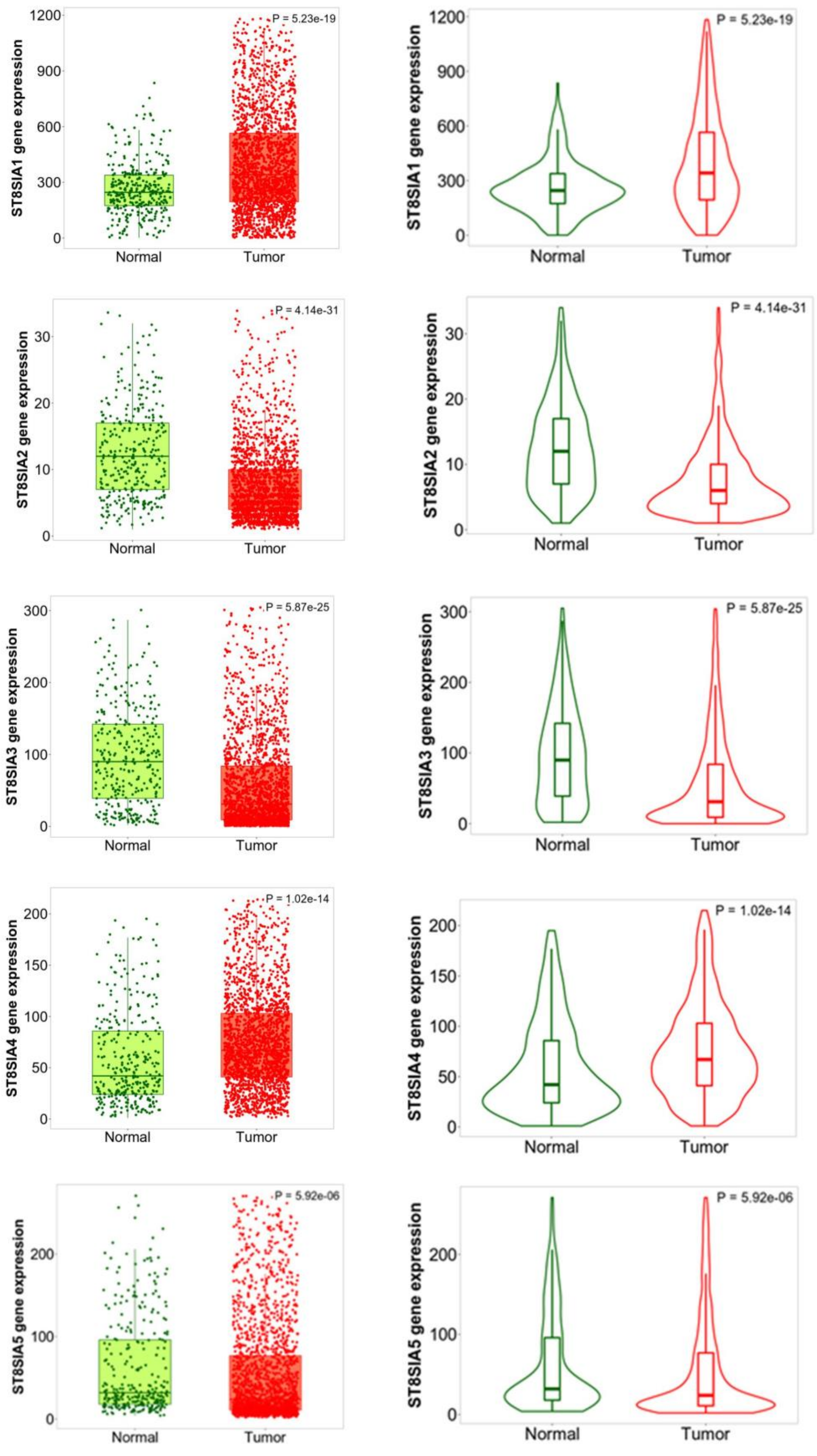

Figure S9: ST8SIA family gene expression in CNS tumors and normal tissue. Boxplots (left) and violin plots (right) of ST8Sia family gene expression in CNS tumors when comparing paired normal and tumor microarray data. Comparisons were performed using the Mann-Whitney U test with a statistical significance threshold set at p value <0.01. Data was obtained from TNMplot (TNMplot.com last access date 03/19/2023).

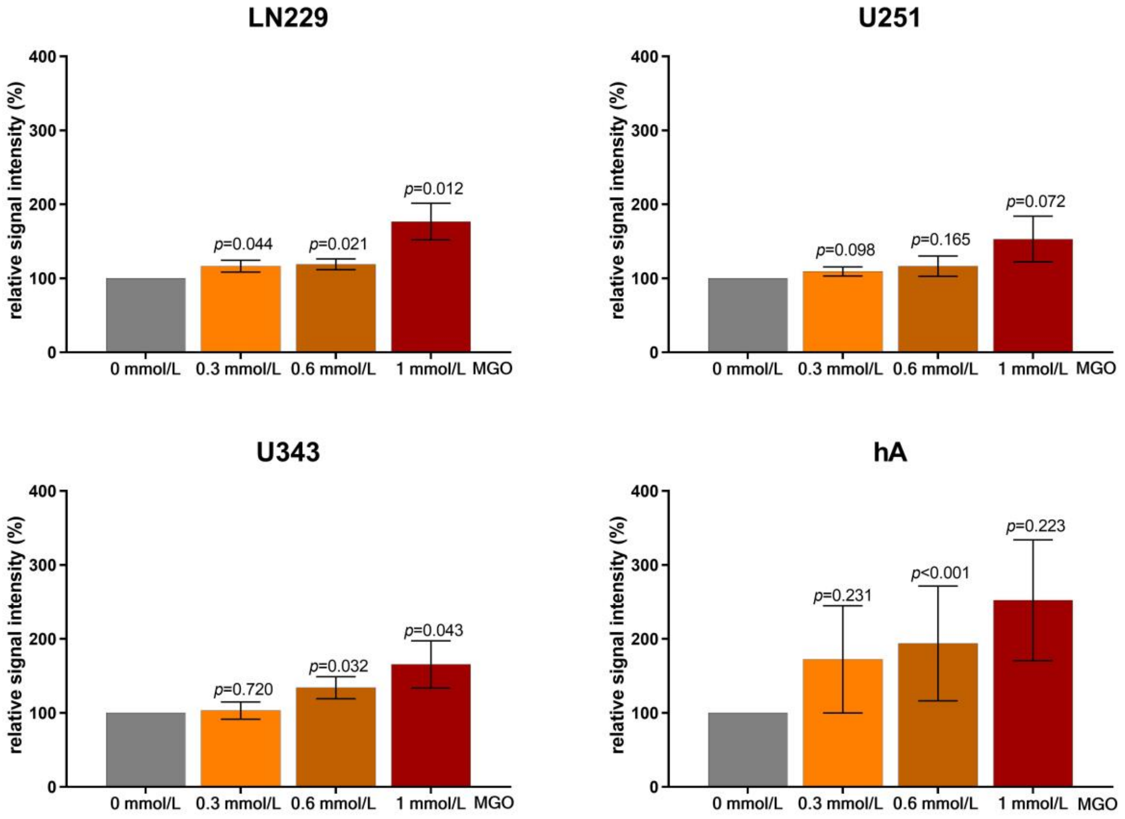

Figure S10: Quantification of Glycation of glioma cell lines and hA. Graphs show representative quantification of the blot, normalized to the untreated cells. GAPDH was used as loading control. Student's t-test was performed for statistical analysis. Graphs represent the means and SD of three independent biological replicates. Western Blot and Quantification of glioma cell lines and hA after glycation has been published previously and is shown here for clarification [3].

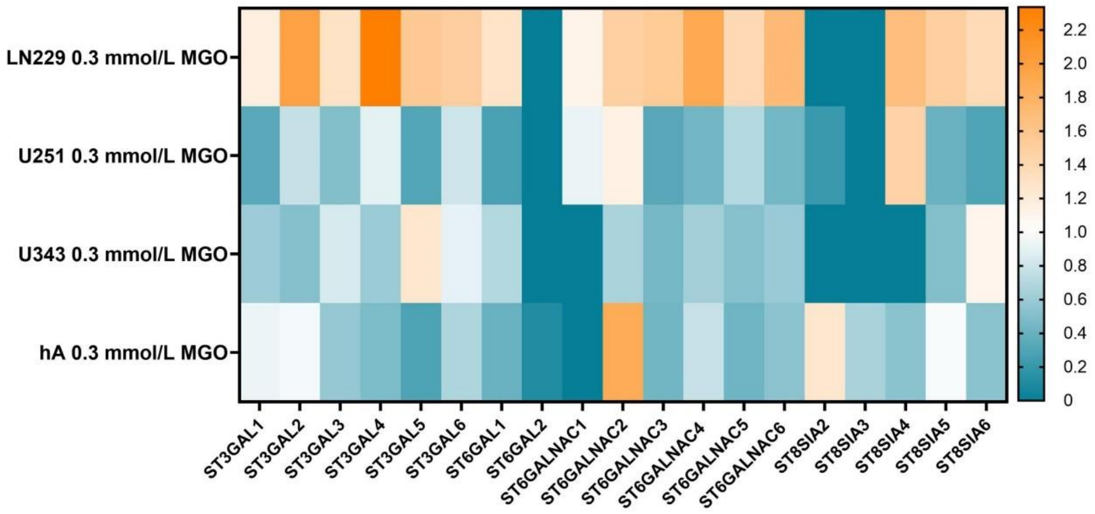

Figure S11: Sialyltransferase expression after glycation. Heatmap of ST mRNA levels in LN229 cells, U251 cells, U343 cells and hA after treatment with 0.3 mmol/L MGO normalized to untreated cells, measured with qPCR.

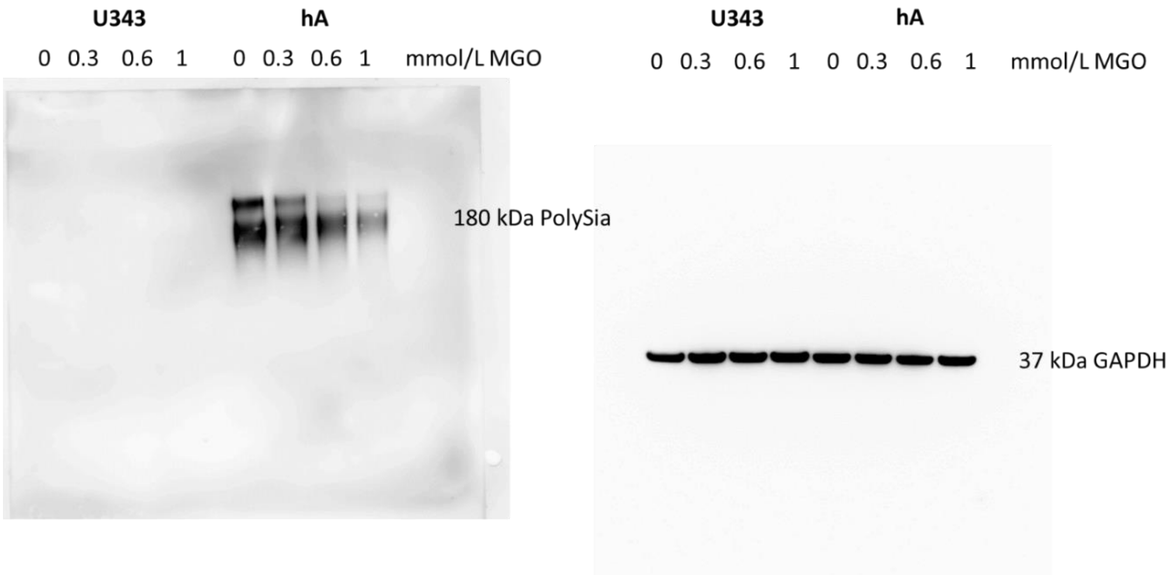

Figure S12: Polysialylation after MGO treatment. Immunoblot of U343 and hA with different MGO concentrations (0.1, 0.3 and 0.6 mmol/L) Polysialylation was detected with PolySia antibody (right). GAPDH was used as loading control left).

Table S2: Baseline data of glioma patients from IHC tissue samples.

Diagnosis	MGMT	IDH	Age	Sex
Glioblastoma	unmethylated	wt	77	m
Glioblastoma	methylated	wt	48	m
Glioblastoma	unmethylated	wt	59	f
Glioblastoma	methylated	wt	84	f
Glioblastoma	unmethylated	wt	55	m

Abbreviations: IDH: isocitrate dehydrogenase; wt: wild-type; MGMT: O-(6)-methylguanine-DNA methyltransferase; f: female; m: male.

References:

1. Ishii, N.; Maier, D.; Merlo, A.; Tada, M.; Sawamura, Y.; Diserens, A.; Van Meir, E.G. Frequent Co-Alterations of TP53, P16/CDKN2A, P14 ARF , PTEN Tumor Suppressor Genes in Human Glioma Cell Lines. *Brain Pathol.* **1999**, *9*, 469–479, doi:10.1111/j.1750-3639.1999.tb00536.x.

2. Patil, V.; Pal, J.; Somasundaram, K. Elucidating the Cancer-Specific Genetic Alteration Spectrum of Glioblastoma Derived Cell Lines from Whole Exome and RNA Sequencing. *Oncotarget* **2015**, *6*, 43452–43471, doi:10.18632/oncotarget.6171.

3. Schildhauer, P.; Selke, P.; Scheller, C.; Strauss, C.; Horstkorte, R.; Leisz, S.; Scheer, M. Glycation Leads to Increased Invasion of Glioblastoma Cells. *Cells* **2023**, *12*, 1219, doi:10.3390/cells12091219.
